# The glycoprotein GPNMB attenuates astrocyte inflammatory responses through the CD44 receptor

**DOI:** 10.1186/s12974-018-1100-1

**Published:** 2018-03-08

**Authors:** Matthew L. Neal, Alexa M. Boyle, Kevin M. Budge, Fayez F. Safadi, Jason R. Richardson

**Affiliations:** 1 Department of Pharmaceutical Sciences, Center for Neurodegenerative Diseases and Aging, Northeast Ohio Medical University, Rootstown, OH 44272 USA; 2 Department of Anatomy and Neurobiology, Northeast Ohio Medical University, Rootstown, OH 44272 USA; 3 Department of Biomedical Sciences, Kent State University, Kent, OH 44240 USA

**Keywords:** Astrocyte, Parkinson’s disease, Neuroinflammation, GPNMB, CD44

## Abstract

**Background:**

Neuroinflammation is one of the hallmarks of neurodegenerative diseases, such as Parkinson’s disease (PD). Activation of glial cells, including microglia and astrocytes, is a characteristic of the inflammatory response. Glycoprotein non-metastatic melanoma protein B (GPNMB) is a transmembrane glycoprotein that releases a soluble signaling peptide when cleaved by ADAM10 or other extracellular proteases. GPNMB has demonstrated a neuroprotective role in animal models of ALS and ischemia. However, the mechanism of this protection has not been well established. CD44 is a receptor expressed on astrocytes that can bind GPNMB, and CD44 activation has been demonstrated to reduce NFκB activation and subsequent inflammatory responses in macrophages. GPNMB signaling has not been investigated in models of PD or specifically in astrocytes. More recently, genetic studies have linked polymorphisms in GPNMB with risk for PD. Therefore, it is important to understand the role this signaling protein plays in PD.

**Methods:**

We used data mining techniques to evaluate mRNA expression of GPNMB and its receptor CD44 in the substantia nigra of PD and control brains. Immunofluorescence and qPCR techniques were used to assess GPNMB and CD44 levels in mice treated with MPTP. In vitro experiments utilized the immortalized mouse astrocyte cell line IMA2.1 and purified primary mouse astrocytes. The effects of recombinant GPNMB on cytokine-induced astrocyte activation was determined by qPCR, immunofluorescence, and measurement of nitric oxide and reactive oxygen production.

**Results:**

Increased GPNMB and CD44 expression was observed in the substantia nigra of human PD brains and in GFAP-positive astrocytes in an animal model of PD. GPNMB treatment attenuated cytokine-induced levels of inducible nitric oxide synthase, nitric oxide, reactive oxygen species, and the inflammatory cytokine IL-6 in an astrocyte cell line and primary mouse astrocytes. Using primary mouse astrocytes from CD44 knockout mice, we found that the anti-inflammatory effects of GPNMB are CD44-mediated.

**Conclusions:**

These results demonstrate that GPNMB may exert its neuroprotective effect through reducing astrocyte-mediated neuroinflammation in a CD44-dependent manner, providing novel mechanistic insight into the neuroprotective properties of GPNMB.

## Background

Parkinson’s disease (PD) is the second most common neurodegenerative disease behind Alzheimer’s disease [1]. It is a chronic, progressive disease with severe motor deficits, including resting tremors, bradykinesia, akinesia, and postural instability. The main factor that contributes to the motor dysfunctions seen in PD is the selective loss of dopamine neurons in the substantia nigra (SN). The major pathogenic mechanisms involved in PD include mitochondrial dysfunction, protein aggregation, oxidative stress, and neuroinflammation [2]. With regard to neuroinflammation, microglia and astrocytes respond to neuronal injury or toxic stimuli by altering cellular morphology and producing increased levels of inflammatory factors, such as pro-inflammatory cytokines. In PD patients and animal models of PD, there is an increased number of activated microglia and astrocytes accompanied by increased release of pro-inflammatory cytokines in the nigrostriatal pathway [3–6]. Pro-inflammatory cytokine signaling in astrocytes leads to propagation of the inflammatory response and can reduce the ability of this cell type to protect neurons from cell death [7].

Glycoprotein non-metastatic melanoma protein B (GPNMB) is a transmembrane glycoprotein named for being highly expressed in a melanoma cell line along with low metastatic properties [8]. Because of the role of GPNMB in osteoblast differentiation and increasing bone mineral deposition, it is also known as osteoactivin [9, 10]. Studies have suggested roles for GPNMB in regulating systemic immune response, including the inhibition of T lymphocyte activation, and reducing macrophage inflammatory response to lipopolysaccharide (LPS) [11, 12]. Similarly, the DBA2J mouse, which expresses a truncated, non-functional GPNMB protein, produces higher levels of inflammatory cytokines following LPS stimulation (Ripoll et al. [11]). In addition to the peripheral immune system, the rat brain ubiquitously expresses GPNMB, including expression in astrocytes [13, 14]. More recently, the spinal cord of amyotrophic lateral sclerosis (ALS) patients was shown to exhibit increased expression of GPNMB compared to control patients, as well as increased GPNMB expression in animal models of both ALS and ischemic injury [13, 15]. Transgenic overexpression of GPNMB led to prolonged survival of the SOD1^G93A^ mouse model of ALS and reduced infarct volume in a reperfusion ischemic injury mouse model [13, 16, 17], suggesting an important role in neuroprotection. With regard to PD, several single nucleotide polymorphisms (SNPs) around the GPNMB gene have been linked to risk for developing PD [18, 19]. However, the mechanisms responsible for these observations are not well established.

GPNMB is capable of signaling through multiple receptors, including CD44. Previous work from the Safadi laboratory demonstrated that GPNMB can directly bind to CD44 in osteoclast precursor cells through docking studies and immunoprecipitation [20]. A similar study from the same lab found that GPNMB can directly bind to CD44 in mesenchymal stem cells [21]. CD44 is a widely expressed hyaluranan receptor with demonstrated roles in cell adhesion and migration [22]. Additionally, CD44 was shown to interact with TLR2 and reduce NFκB signaling, suggesting a role in reducing the inflammatory response in bone marrow macrophages [23]. In addition to macrophages, CD44 was demonstrated to be present in fetal and adult astrocytes [24]. Some evidence suggests that CD44 is involved in exacerbating cerebral ischemia possibly through the production of the pro-inflammatory cytokine IL-1β, with CD44-deficient mice significantly protected from cerebral ischemia and reduced levels of IL-1β [25]. However, another group found that CD44-deficient mice could not resolve the inflammatory response in the lung, with defective clearance issues and TGFβ activation, indicating the importance of this receptor in resolving inflammation [26].

Currently, there are no studies investigating the role of GPNMB signaling in astrocytes, the most abundant glial cell in the brain. Because of the known functions of GPNMB in dampening the immune response, we hypothesized that GPNMB would attenuate the astrocyte inflammatory response to cytokines in a CD44-dependent manner. Our data indicate prominent expression of CD44 and GPNMB in astrocytes that is increased in an animal model of PD. Further, treatment with the extracellular fragment of GPNMB potently reduced the ability of pro-inflammatory cytokines to activate astrocyte cultures. Importantly, this effect was blocked in astrocytes isolated from mice lacking CD44. Together, these data provide a strong mechanistic model for the role of GPNMB and CD44 in astrocytes and suggest that GPNMB may represent a viable therapeutic approach for reducing neuroinflammation in diseases such as PD.

## Methods

### Reagents

DMEM, MEM media, fetal bovine serum (FBS), l-glutamine, IR-dye tagged secondary antibodies, penicillin, streptomycin, and other cell culture reagents were purchased from Invitrogen (Gaithersburg, MD). Recombinant GPNMB (> 90% purity), was purchased from R&D Systems (Cat #: 255-AC-050). The primary mouse monoclonal antibody for inducible nitric oxide synthase (iNOS) (Cat #: sc-7271) and the mouse monoclonal arginase-1 (Cat #: sc-271430) antibody was ordered from Santa Cruz Biotechnology Inc. (Santa Cruz, CA). The chicken polyclonal glial firbrillary acidic protein (GFAP) antibody (Cat. #: ab4674) was obtained from Abcam (Cambridge, MA). The rabbit polyclonal GPNMB (Cat. #: bs-2684r) and rabbit polyclonal CD44 antibody (Cat. #: bs-0521r) was purchased from Bioss (Woburn, MA). 1-Methyl-4-phenyl-1,2,3,6-tetrahydropyridine (MPTP) and all other reagents were obtained from Sigma-Aldrich (St. Louis, MO), unless otherwise indicated.

### Data mining of GPNMB and CD44 gene expression in human tissue

Human gene expression data for GPNMB and CD44 was obtained from the NCBI gene expression obmnibus (GEO), which is a public repository of gene expression profiles from high-throughput microarray experiments with the option to data mine for specific genes of interest [27]. Data was mined from two studies that collected the substantia nigra of PD patients (total *n* = 22 males and *n* = 9 females) along with age-matched controls (total *n* = 10 males and *n* = 7 females), which were run on the Affymetrix gene chip microarray [28–30]. The GEO accession number for the referenced studies are GDS2821 and GDS3128, with the GPNMB target ID numbers 1554018_at for GDS2821 and 201141_at for GDS3128, respectively, and the CD44 target ID numbers 217523_at for GDS2821 and 204490_s_at for GDS3128. The gene expression results in GEO are expressed as arbitrary units in the GEO due to different preparations of samples and analysis, but signify levels of gene expression. To allow examination of relative expression for these genes across the two different datasets, the expression values were normalized to controls and analyzed by Welch’s *t* test which was to account for the different sample number and variances.

### Cell culture and animal studies

Immortalized mouse astrocytes (IMA2.1) was grown in MEM media supplemented with 10% FBS, penicillin (100 U/ml), and streptomycin (100 μg/ml), according to previous literature [31]. Primary mouse astrocytes were obtained from whole brain homogenate of 0–3 day mouse pups. Astrocytes were collected, isolated, and maintained according to previous methods (Gordon et al. [32]) using differential adherence and immune-selecting the microglia out of the mixed glial cultures and using DMEM media (10% heat-inactive FBS, 100 U/ml penicillin, 100 μg/ml streptomycin, glutamine 2 mM) to grow the cells.

All animal procedures were approved by the Northeast Ohio Medical University’s Institutional Animal Care and Use Committee (IACUC) and conducted according to the NIH Guide for the Care and Use of Laboratory Animals. C57 BL/6J mice were obtained from Jackson laboratory and housed under a 12-h light cycle in an AAALAC-approved climate-controlled animal facility (22 ± 1 °C) with food and water available ad libitum. Males were used for all studies. All mice were pre-screened for normal weight and behavior before randomly assigning animals to experimental groups. Investigators involved with data collection and analysis were not blinded to group allocation. 1-Methyl-4-phenyl-1,2,3,6-tetrahydropyridine (MPTP) was administered by intraperitoneal injection at a dose of 10 mg/kg once every 2 h for a total of four injections. Mice were sacrificed 2 or 7 days post injection. This acute MPTP treatment paradigm is commonly used to study neuroinflammation and dopaminergic neurononal cell death in the substantia nigra [33, 34].

### Quantitative PCR

Primary mouse astrocytes or IMA cells were seeded into 24-well plates at 70,000 cells per well. Treatments were performed in either MEM for the IMA cells or DMEM media for the primary mouse astrocytes supplemented with 10% FBS, penicillin (100 U/ml), streptomycin (100 μg/ml), and 2 mM l-glutamine. Cells were treated for 6 h with an inflammatory cytokine mix (CM) consisting of TNFα (5 ng/mL), IL-1β (5 ng/mL), and IFNγ (10 ng/mL) to induce an inflammatory response. Cells were co-treated with CM along with rGPNMB (50 ng/mL) for 6 h. After treatment, treatment media was removed and IBI isolate was added to the wells to lyse the cells (IBI Scientific, Peosta, IA). RNA was isolated according to the manufacturer’s directions.

RT-PCR was performed using a cDNA synthesis system (Bimake, Houston, TX) to convert the RNA into cDNA. Expression levels were determined using real-time PCR with Bimake RT2 SYBR Green master mix and previously published primer sets *Nos2* For-TCACGCTTGGGTCTTGTT and Rev-CAGGTCACTTTGGTAGGATTT, *Il-6* For-TCCATCCAGTTGCCTTCTTG and Rev-ATTGCCATTGCACAACTCTTTT, *Arginase-1* For-GGACCTGGCCTTTGTTGATG and Rev-AGACCGTGGGTTCTTCACAATT, *Igf-1* For-CGCCTCATTATCCCTGCCCACCA and Rev-GCCATAGCCTGTGGGCTTGTTGAA, *Gpnmb* For-AATGGGTCTGGCACCTACTG and Rev-GGCTTGTACGCCTTGTGTTT, and *Cd44* For-GAATTCTGCGCCCTCGGTT and Rev-CTGCCTCAGTCCGGGAGATA. For normalization of each sample, the mouse genes *Gapdh* For-TGAAGCAGGCATCTGAGGG and Rev-CGAAGGTGGAAGAGTGGGAG *Rpl13a* For-CTGTGAAGGCATCAACATTTCTG and Rev-GACCACCATCCGCTTTTTCTT were used as the housekeeping genes. The amount of each template was optimized empirically to maximize efficiency without inhibiting the PCR reaction. According to the manufacturer’s guidelines, dissociation curves and melting curves were run to ensure that single amplicon peaks were obtained without any non-specific amplicons. The results are reported as fold change in gene expression, which was determined using the ΔΔ^Ct^ method using the threshold cycle (Ct) value for the housekeeping gene and for the respective gene of interest in each sample.

### Immunocytochemistry and immunohistochemistry

Immunocytochemistry was performed as previously described [35], cells were plated onto 8-well chamber slides coated with 0.1% poly-d-lysine. After cells were treated for 10 h, 4% formaldehyde was used to fix the cells for 30 min. The cells were washed with PBS buffer. Then, blocking buffer containing 2% bovine serum albumin (BSA), 0.2% Triton X-100, and 0.2% Tween-20 was added to the wells for 1 h. The cells were incubated with primary antibodies in 2% BSA at 4 °C overnight. Next, an Alexafluor dye-conjugated secondary antibody in 2% BSA was added and incubated at room temperature on a shaker for 1 h. After washing, coverslips were added to the slides using Fluorshield GOLD mounting media containing DAPI nuclear stain. Cells were imaged using an Olympus FSX imaging instrument.

Histology was performed as previously described [34, 36]. Briefly, brains were drop-fixed in 4% paraformaldehyde for 7 days. After 7 days, brains were washed with PBS and placed into a 30% sucrose solution for at least 24 h. The brains were then flash frozen with crushed dry ice and were sectioned at 40 μm on a Thermo scientific Microm HM 450 sliding arm microtome with dry ice, and the sections were placed into Cryosolution (30% sucrose, ethylene glycol, and PBS). Sections were then washed with PBS and permeabilized with blocking buffer (2% BSA, 0.1% Triton X-100, and Tween) for 1 h at room temperature. Antibodies directed to the protein of interest were then incubated with the sections overnight at 4 °C in 2% BSA. After washing several times with PBS, the sections were incubated with Alexafluor dye-conjugated goat secondary antibodies (1:2000) for 1 h at room temperature. After washing with PBS, sections were then mounted on slides using the Molecular Probes ProLong GOLD anti-fade mounting medium containing DAPI stain according to the manufacturer’s instructions. Sections were imaged using the Leica TCS SPE confocal microscope.

Quantification of protein immunofluorescence was performed for both immunocytochemistry and immunohistochemistry by using ImageJ analysis software. For immunocytochemistry, 4–6 separate fields were imaged for each treatment and repeated for a total of at least two experimental replicates. For immunohistochemistry, at least three fields were chosen for each section and three different sections for each treatment. At least two animals were analyzed for each treatment.

### Reactive oxygen species and nitric oxide quantification

The fluorescent dye CM-H_2_DCFDA was used to determine intracellular reactive oxygen species (iROS). IMA2.1 or primary mouse astrocytes (PMA) cells were seeded out into black-walled 96-well plates and incubated with the dye in HBSS for 1 h. Next, the cells were washed thrice with HBSS media to remove any residual dye outside of the cells. The cells were then treated with CM, rGPNMB, or co-treated with both in HBSS. Fluorescent intensity was measured once every 30 min for 6 h on a SpectraMax M5 plate reader. The fluorescent values for four wells were averaged for each plate.

The Griess colorimetric assay was chosen for the nitric oxide (NO) determination. IMA2.1 or PMA cells were seeded into 96-well plates and treated with no treatment, CM, rGPNMB, or co-treated with both for 24 h. Following the treatment, the media was collected and run using a standard curve from the kit according to manufacturer’s directions. The measured absorbance was compared to the standard curve to give total concentration of media nitrite. Values were averaged for four wells per plate to produce one biological replicate.

### Data analysis

Data analysis was performed using the Prism 5.0 software package (GraphPad Software, San Diego, CA). The data were first analyzed using either one- or two-way ANOVA and then Tukey’s posthoc test was performed to compare all treatment groups. Differences of *p* < 0.05 were considered statistically significant. The Student’s *t* test was used when two groups were being compared, with the exception of the human gene expression data that were analyzed by Welch’s *t* test. All in vitro experiments were performed with at least three replicates per experiment from at least two independent experiments.

## Results

### Increased GPNMB and CD44 levels in human PD patients and a mouse MPTP model

**Fig. 1:**
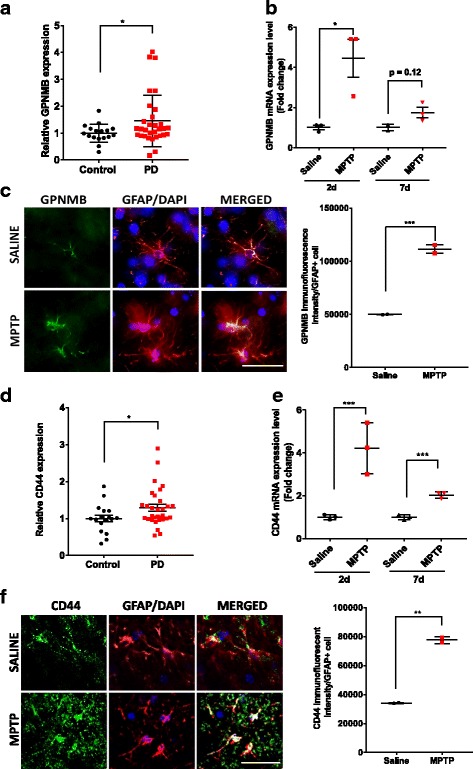
Increased GPNMB and CD44 levels in PD patients and an acute MPTP mouse model. **a** Gene expression of GPNMB is increased in the substantia nigra of PD patients (*n* = 22 males and *n* = 9 females) compared to age-matched controls (*n* = 10 males and *n* = 7 females). GEO numbers were normalized to 1 to generate relative expression values and allow comparison between datasets. **b** GPNMB gene expression in the mouse striatum by qPCR following an acute injection of MPTP in mice and sacrificed at 2 and 7 days post injection. **c** Representative images of immunofluorescent confocal microscopy of GPNMB (green), GFAP (red), and DAPI nuclear stain (blue) at × 40 magnification following an acute injection of saline or MPTP, scale bar and 50 μm. **d** Gene expression of CD44 in human PD patients examined by microarray on the GEO demonstrating a significant increase in CD44 gene expression in the SN of PD patients. **e–f** CD44 gene expression and protein levels in the mouse striatum by **e** qPCR and immunofluorescent confocal microscopy of CD44 (green), GFAP (red), and DAPI nuclear stain (blue) at **f** × 40 magnification following an acute injection of Saline or MPTP, scale bar and 50 μm. Asterisks denote statistically significant differences between PD and age-matched controls or MPTP and saline controls (*p < 0.05, **p < 0.01, and ***p < 0.001)

Recently, GPNMB was identified as a potential risk gene for the development of PD, and one study found that a particular SNP at the 7p15 chromosomal region associated with altered PD risk led to increased GPNMB expression [18, 19]. Therefore, we hypothesized that GPNMB would be increased in the substantia nigra of PD patients compared to age-matched controls. The gene expression omnibus (GEO) is a repository for functional genomics data run by the National Center for Biotechnology Information (NCBI). Investigators can upload high-throughput genomics data and it is open to the public. Two different studies generated gene expression profiles by microarray in the SN of PD patients (*n* = 22 males and *n* = 9 females) compared to controls (*n* = 10 males and *n* = 7 females) and uploaded the results to the GEO [28, 29, 37]. We examined the gene expression of GPNMB in these samples and found that GPNMB expression is significantly higher in the PD patients compared to that in the control, with over 45% increased expression (Fig. 1a).

Next, we sought to determine the expression of GPNMB in an animal model of PD, utilizing an acute MPTP injection paradigm comprised of four total injections once every 2 h and sacrificed the mice either 2 or 7 days later. We found a significant fourfold increase in GPNMB mRNA expression in the striatum 2 days following the MPTP treatment. Increased gene expression was maintained through 7 days following injection, but this did not quite reach statistical significance (*p* = 0.12) (Fig. 1b). Using immunofluorescent confocal microscopy, we found that GFAP-positive cells in the striatum of MPTP-treated mice contained around twofold higher levels of GPNMB compared to those of saline-treated animals (Fig. 1c).

Astrocytes express CD44 under basal conditions and increase in response to an inflammatory stimulus [38, 39]. However, there are no published reports on the levels of CD44 in the nigrostriatal pathway in PD patients or animal models of PD. Therefore, we first examined CD44 expression from the same microarray study from the GEO that we examined GPNMB levels. We found that CD44 gene expression in the substantia nigra of PD patients was significantly increased compared to age-matched controls (Fig. 1d). Next, we examined CD44 expression in the striatum of MPTP-treated mice. Similar to GPNMB, MPTP treatment significantly increased CD44 expression fourfold over saline-treated animals 2 days after injection, and this expression was still significantly increased 7 days later (Fig. 1d).

To confirm our mRNA data, we used immunofluorscence to visualize the increased CD44 protein level in the SN. MPTP treatment resulted in increased overall CD44 protein levels in the SN, along with an increase in GFAP (data not shown). Utilizing higher magnification microscopy, we were able to demonstrate that the GFAP-positive cells in the SN express CD44 under basal conditions and that MPTP treatment increases CD44 immunofluorescent staining approximately twofold higher compared to saline-treated animals (Fig. 1e). Consistent with previous results in mouse astrocytes [40], we found that CD44 protein was throughout the astrocyte, including the cell processes. The MPTP-induced CD44 protein level did not appear to be exclusive to GFAP-positive cells, which would indicate that MPTP might increase CD44 in microglial cells or dopaminergic neurons, both of which have been found to express CD44 to some degree [40, 41]. Taken together, these data demonstrate increased GPNMB and CD44 gene expression in the substantia nigra of PD patients and in animal models of PD, with increased CD44 protein levels in GFAP-positive astrocytes following MPTP treatment.

### GPNMB signaling in astrocyte cultures

**Fig. 2:**
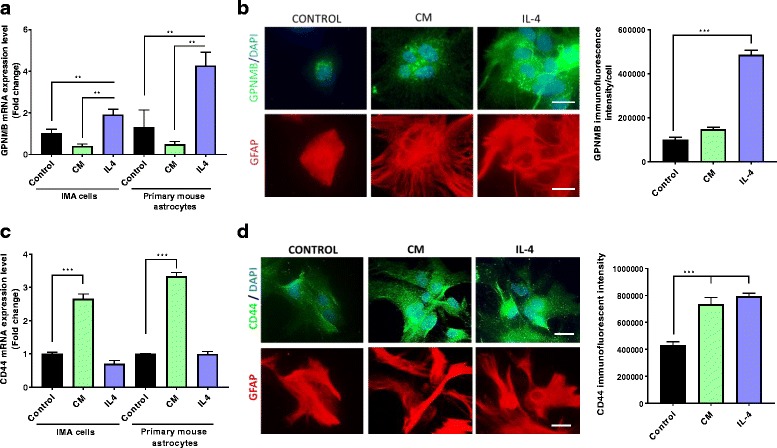
Increased GPNMB and CD44 in cultured astrocytes exposed to different stimuli*.* **a–b** GPNMB gene expression (**a**) in cultured IMA2.1 cells and PMAs by qPCR, and protein levels (**b**) by immunofluorescent microscopy in PMAs (GPNMB in green, DAPI in blue, and GFAP in red). **c** CD44 gene expression in IMA2.1 cells and PMAs treated with or without the inflammatory cytokine mix (CM) or recombinant IL-4. **d** Immunofluorescence images for CD44 (green) protein levels in GFAP-positive (red) primary mouse astrocytes following CM or IL-4 treatment (scale bar 20 μm). Asterisks denote statistically significant differences between CM or IL-4 treatment and control (**p < 0.01 and ***p < 0.001)

Based on the observance of increased CD44 levels in astrocytes following MPTP treatment, we utilized a pure immortalized mouse astrocyte cell line (IMA2.1) and isolated primary postnatal (0–3 days old) mouse astrocytes (PMAs) to determine GPNMB and CD44 expression. Previously, Yu and co-workers found that treatment of mesenchymal stem cells with lipopolysaccharide (LPS) and interferon gamma (IFNγ) did not significantly increase GPNMB expression, but treatment with the anti-inflammatory cytokine IL-4 significantly increased GPNMB expression [21]. Therefore, we sought to determine if cultured astrocytes would respond in a similar fashion by treating cultured astrocytes with an inflammatory cytokine mixture (TNFα, IL-1β, and IFNγ) or the anti-inflammatory cytokine IL-4. The IMA2.1 cell line and PMAs expressed GPNMB basally and treatment with the anti-inflammatory cytokine IL-4 induced a significant increase in gene expression, whereas treatment with an inflammatory cytokine mixture (TNFα, IL-1β, and IFNγ) did not significantly increase GPNMB expression (Fig. 2a). This result was validated using fluorescence microscopy, with the primary mouse astrocytes expressing the GPNMB protein and IL-4 treatment increasing the protein level (Fig. 2b).

Next, we measured CD44 levels in both the IMA2.1 and PMA cells. Consistent with previous results, both the IMA2.1 and PMAs expressed CD44 and the inflammatory cytokine mix (CM) significantly increased CD44 gene expression (Fig. 2c) and protein level (Fig. 2d). Gene expression of CD44 was not altered with IL-4 treatment; however, the protein level was significantly increased with IL-4 treatment. We hypothesize that the CD44 gene expression was increased after the 6 h timepoint, leading to CD44 protein increases at the later timepoint. However, it is possible that IL-4 increased CD44 protein levels by reducing protein degradation, which remains to be established. Together, these results demonstrate that the IMA2.1 cell line and PMAs express both GPNMB and CD44 and that the inflammatory status of these cells alters the expression of GPNMB and CD44. Thus, the GPNMB-CD44 signaling pathway could potentially be a mechanism used by the cell to resolve the inflammatory response, with increased CD44 following a pro-inflammatory stimulus and increased GPNMB levels following an anti-inflammatory stimulus.

### Recombinant extracellular fragment of GPNMB reduced inflammatory mediators and increased anti-inflammatory factors in cultured astrocytes

**Fig. 3:**
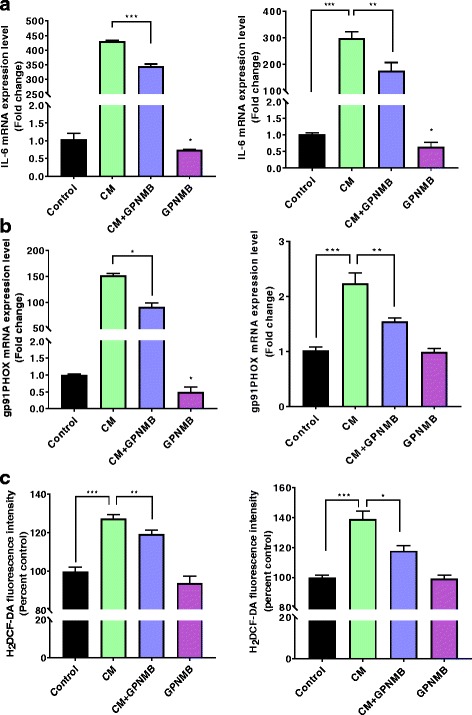
GPNMB reduces inflammatory mediators in cultured astrocytes*.* **a–b** Gene expression of the inflammatory factors IL-6 (**a**) and gp91phox (**b**) in IMA cells (left panel) and PMAs (right panel). **c** H_2_DCF-DA fluorescence intensity measuring intracellular ROS generation in IMA (left panel) and PMA (right panel) cells. Asterisks denote statistically significant differences between the different treatment groups or compared to the control (*p < 0.05, **p < 0.01, and ***p < 0.001)

The extracellular region of the transmembrane GPNMB is cleaved by the metalloprotease ADAM10 leading to a soluble extracellular fragment of GPNMB. To determine whether addition of the recombinant extracellular fragment of GPNMB (rGPNMB) could attenuate the inflammatory response from cytokine mix (CM) treatment in the cultured astrocytes, we treated astrocyte cultures with CM and measured pro- and anti-inflammatory factors. CM treatment resulted in a significant increase in the gene expression of the inflammatory factors IL-6 and gp91phox in both the IMA2.1 cells and the PMAs (Fig. 3a, b). rGPNMB co-treatment significantly reduced the CM-induced IL-6 gene expression in both cultures (Fig. 3a), and rGPNMB treatment alone significantly reduced the basal level of this inflammatory cytokine in both the IMA2.1 cells and PMAs (Fig. 3a). The rGPNMB treatment significantly reduced the CM-induced gene expression of gp91phox in both IMA2.1 cells and PMAs, but at the timepoint measured, the rGPNMB treatment alone reduced basal gene expression of gp91phox in IMA2.1 cells by almost 40%, but gp91phox gene expression was not altered (Fig. 3b). Gp91phox is the catalytic subunit of the NADPH oxidase, and increased gp91phox leads to increased production of reactive oxygen species (ROS). rGPNMB co-treatment with CM significantly reduced the generation of ROS in both IMA2.1 cells (Fig. 3c, left panel) and PMAs (Fig. 3c, right panel). Overall, the IMA2.1 cells exhibited greater responses to both the CM and rGPNMB treatment alone, which could be due to multiple factors. The end product of ROS production between the two cultures was very similar, and the difference in gp91phox gene expression in the two cultures could be due to the specific timepoint measured.

**Fig. 4:**
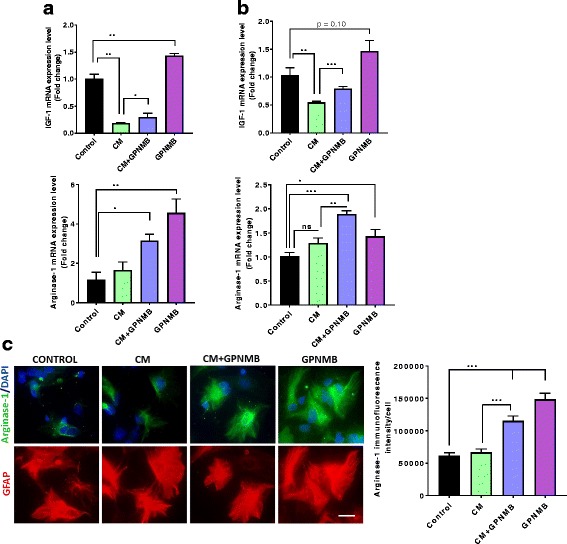
rGPNMB induces anti-inflammatory factors IGF-1 and arginase-1 in cultured astrocytes*.* **a–b** Gene expression of the anti-inflammatory factors IGF-1 (top panel) and arginase-1 (bottom panel) in IMA cells (**a**) and PMAs (**b**). **c** Representative immunofluorescent images and quantification for arginase-1 in PMAs treated with CM alone, GPNMB alone or co-treated with CM and GPNMB (scale bar 20 μm). Asterisks denote statistically significant differences between the different treatment groups or compared to the control (*p < 0.05, **p < 0.01, and ***p < 0.001)

To determine the effect of rGPNMB on anti-inflammatory factors, we assessed the gene expression of arginase-1 and insulin growth factor-1 (IGF-1). CM treatment significantly reduced the expression of IGF-1, but did not significantly change arginase-1 in either the IMA2.1s or the PMAs (Fig. 4a, b). rGPNMB alone significantly increased gene expression of arginase-1 and IGF-1 in both the cultures. Co-treatment with rGPNMB significantly attenuated the CM-induced loss of IGF-1 gene expression. To confirm the gene expression results, arginase-1 fluorescent microscopy showed significantly increased protein level with rGPNMB treatment alone and co-treated with CM (Fig. 4c). These data demonstrate that rGPNMB can reduce the CM-induced inflammatory response in cultured astrocytes and promote more of an anti-inflammatory phenotype.

### Attenuation of inflammatory cytokine-induced nitric oxide production following rGPNMB treatment

**Fig. 5:**
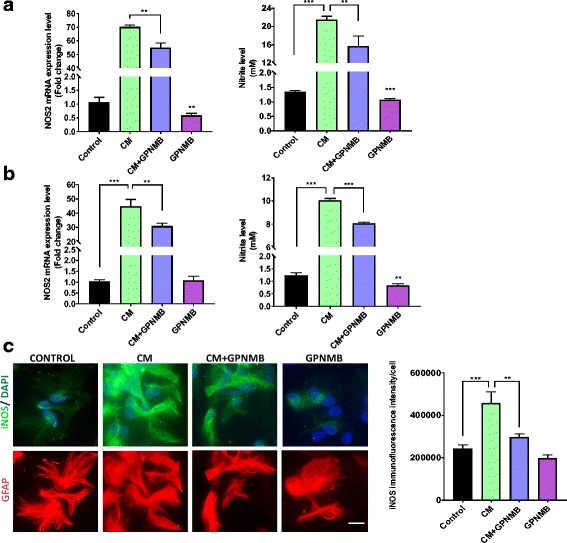
GPNMB attenuated cytokine-induced reactive nitrogen species production*.* **a–b** Gene expression of NOS2 (left panel) and cell culture supernatant nitrite levels (right panel) from IMA cells (**a**) and PMAs (**b**) treated with either CM, GPNMB, or co-treated with both. **c** Representative immunofluorescent images and quantification for iNOS protein levels in PMAs treated with CM, GPNMB, or co-treated with both (scale bar 20 μm). Asterisks denote statistically significant differences between the different treatment groups or compared to the control (*p < 0.05, **p < 0.01, and ***p < 0.001)

Astrocytes produce higher levels of nitric oxide (NO) when exposed to an inflammatory stimulus [42] and NO can combine with ROS to form damaging peroxinitrite (ONOO^−^). The enzyme responsible for NO generation, iNOS, competes with arginase-1 for l-arginine. With rGPNMB reducing the cytokine-induced ROS generation and increasing arginase-1 levels, we next sought to examine whether rGPNMB can attenuate the reactive nitrogen species production in a similar manner. Treatment of the IMA2.1 and the PMAs with CM produced significantly increased gene expression of iNOS (Fig. 5a, b, left panel) and secreted levels of NO, by measuring the levels of nitrite in conditioned media (Fig. 4b, c). Similar to the results for ROS, co-treatment of rGPNMB significantly reduced the gene expression of iNOS, and attenuated the release of NO into the media (Fig. 5a, b).

To confirm the iNOS gene expression results using fluorescent microscopy to measure, the protein level of iNOS in primary mouse astrocytes. We found that the protein levels corresponded to the gene expression results, with CM significantly increasing iNOS protein level and rGPNMB co-treatment attenuating this increase (Fig. 4c). Together, these results demonstrate for the first time that GPNMB can regulate ROS and NO production in cultured astrocytes.

### CD44 deletion abolishes GPNMB-induced reduction of astrocyte inflammatory response in cultured astrocytes

**Fig. 6:**
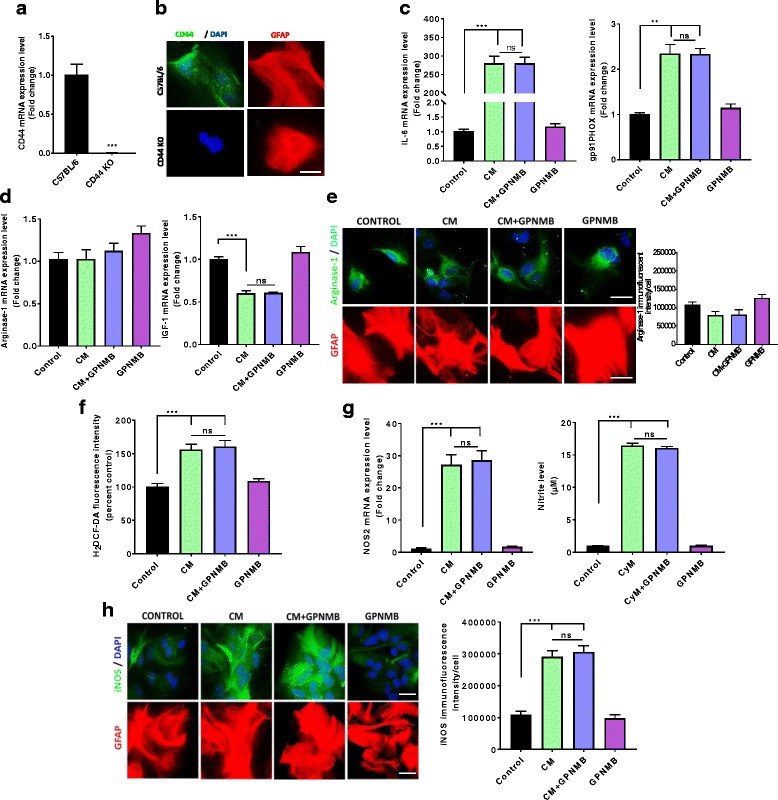
CD44 KO attenuates the GPNMB-induced reduction of cultured astrocyte inflammatory response*.* **a**–**b** Isolated CD44 KO PMAs do not have CD44 gene expression (**a**) or any visible CD44 protein levels (**b**) by immunofluorescent imaging (scale bar 20 μm). **c** Gene expression of the inflammatory factors IL-6 (left panel) and gp91phox (right panel) by qPCR in CD44 KO PMAs. **d** Gene expression of the anti-inflammatory factors arginase-1 (left panel) and IGF-1 (right panel) by qPCR in CD44 KO PMAs. **e** Representative immunofluorescent images for arginase-1 in CD44 KO PMAs treated with CM, rGPNMB or co-treated with both (scale bar 20 μm). **f** H_2_DCF-DA fluorescence intensity measuring intracellular ROS generation in CD44 KO PMAs. **g**–**h** rGPNMB treatment failed to attenuate CM-induced NOS2 gene expression (**g**, left panel), supernatant nitrite levels (**g**, right panel), and iNOS protein levels (**h**) in CD44 KO PMAs. Asterisks denote statistically significant differences between the different treatment groups or compared to the control (*p < 0.05 and ***p < 0.001, not statistically significant groups are represented with ns)

Because increased CD44 expression occurs in astrocytes following an inflammatory stimulus, and GPNMB can signal through CD44, we sought to determine whether the anti-inflammatory effect of rGPNMB in cultured astrocytes is CD44-mediated. Primary astrocytes isolated from CD44 knockout mice did not exhibit CD44 expression, as determined by qPCR and fluorescent microscopy (Fig. 6a, b). CD44 KO astrocytes responded similarly to C57BL/6J astrocytes with increased IL-6 and gp91phox (Fig. 6c). However, unlike the wild-type astrocytes, rGPNMB co-treatment had no effect on the CM induction of IL-6 and gp91phox in the CD44 KO astrocytes (Fig. 6c). The gene expression of arginase-1 and IGF-1 were also unaffected in these CD44 KO astrocytes, although rGPNMB treatment alone still showed a non-statistically significant increase in arginase-1 (Fig. 6d). We then used fluorescent microscopy of arginase-1 to confirm the gene expression results and found that there was no change in arginase-1 protein level with rGPNMB compared to control in the CD44 KO astrocytes (Fig. 6e). These data indicate that the GPNMB attenuation of CM induction of key inflammatory mediators is CD44 receptor-dependent, with CD44 genetic knockouts unable to respond in a similar fashion to the IMA2.1 cell line and C57BL/6J cultured astrocytes. We next determined ROS generation (Fig. 6f), iNOS gene expression (Fig. 6g, left panel), secreted nitrite levels (Fig. 6g, right panel), and iNOS protein levels (Fig. 6h) following co-treatment with CM and rGPNMB. All of these factors showed similar results, with rGPNMB failing to attenuate the CM-induced increase in the CD44 KO astrocytes. The data demonstrate that rGPNB attenuation of the CM-induced production of these factors in astrocytes is CD44-dependent.

## Discussion

There is increasing evidence indicating that astrocytes contribute to neurodegenerative diseases such as Alzheimer’s disease and PD, through increased production of inflammatory cytokines and damaging free radicals that could lead to neuronal death [7, 43, 44]. Therefore, discovery of factors that can modulate astrocyte activation and inflammatory response may represent a novel target for therapeutic intervention. Here, we report that samples from PD patients expressed higher levels of GPNMB in the substantia nigra, the region of the brain with the neuronal loss found in PD, compared to age-matched controls. Further, a significant increase in GPNMB gene expression was observed in the striatum following MPTP treatment. We next identified a role for GPNMB, signaling through CD44, in dampening the response of astrocytes to an inflammatory stimulus. Together, our data demonstrate a significant role for GPNMB and CD44 in the neuroinflammatory process in astrocytes, suggesting that administration of GPNMB may represent a novel therapeutic strategy for reducing neuroinflammation.

Several recent studies have found an association between a SN around the GPNMB gene and an altered risk for developing PD. The International Parkinson’s Disease Genetics Consortium (IPDGC) found that SNP rs156429 in the 7p15 chromosomal region led to decreased risk of developing PD along with increased methylation of CpG sites proximal to the GPNMB gene [18, 45]. Murthy and co-workers further explored the Chr7p15.3 genetic locus that is linked to altered risk for PD and found that the top risk SNP in this region (rs199347) led to increased GPNMB expression in multiple brain regions, indicating that the GPNMB gene could potentially alter risk for PD [19]. This could be population specific, because a cohort examining a Chinese population found that the rs156429 was not associated with PD [46]. Here, we found that GPNMB gene expression was significantly increased 2 days following the last injection of MPTP, but decreased toward control levels 7 days after MPTP administration. This finding is consistent with the findings that 24–48 h post-MPTP represents the peak of inflammatory response in the striatum [47, 48] and precedes dopamine neuron loss in this model [49]. The increased GPNMB expression following MPTP is found prominently in GFAP-positive activated astrocytes in the striatum, similar to that observed in astrocytes and motor neurons of the spinal cord of the SOD1^G93A^ mouse model of ALS [15]. As such, GPNMB expression may be controlled through the inflammatory status of the CNS immune cells, as has been observed in cultured macrophages [21]. The data presented here, along with the epidemiological data linking GPNMB and PD, indicate that GPNMB may contribute to the inflammatory process observed in PD and could represent an early indicator or response to damage in the nigrostriatal system.

Because GPNMB has demonstrated the ability to dampen an inflammatory response in T lymphocytes and macrophages, along with the knowledge that astrocytes upregulate CD44 following an inflammatory stimulus [50, 51], we explored the expression of GPNMB in cultured astrocytes following either a pro- or anti-inflammatory stimulus. In IMA2.1-immortalized mouse astrocytes and PMAs, CM did not alter GPNMB expression, but treatment of cells with the anti-inflammatory cytokine IL-4 resulted in significantly enhanced GPMNB expression. The lack of GPNMB increase with the inflammatory cytokines suggest that the increased GPNMB that we found in vivo could be propagated through different signaling factors that are involved in neuroinflammation or neurodegeneration. These data suggest that the IL-4-mediated increased GPNMB may be involved in the resolution of inflammation as part of the newly described M2 astrocyte pathway [44] and are consistent with our previous work demonstrating that anti-inflammatory factors can elicit higher expression of GPNMB in macrophages [21]. Further support for this hypothesis is provided by the observation that co-treatment with the recombinant GPNMB (rGPNMB) significantly reduced the production of the inflammatory factors IL-6 and gp91phox along with ROS and NO production, while significantly increased the anti-inflammatory factors arginase-1 and IGF-1. Additionally, treatment of the cultured astrocytes with rGPNMB alone also significantly increased both arginase-1 and IGF-1 gene expression, and the protein level of arginase-1. These data further support a role for GPNMB signaling in shifting the inflammatory status of cultured astrocytes toward an anti-inflammatory phenotype, with increased expression of inflammation-resolving factors. This is the first study to report that the transmembrane glycoprotein GPNMB can attenuate inflammatory cytokine-induced inflammatory response in cultured astrocytes. It should be noted that microglia also express CD44, and gene expression of this receptor is increased with microglial activation [52]. Our laboratory is currently exploring how GPNMB signaling through the CD44 receptor could affect microglial activation phenotype (M1 vs. M2) and crosstalk between microglia and astrocytes in PD models.

GPNMB can signal to various cell types through several different receptors, including the Na^+^/K^+^ ATPase, αβ-integrins, FGFR1, heparin sulfate proteoglycans, and CD44 [21, 53–56]. Previous studies have demonstrated that CD44 expression is increased in cultured astrocytes and astrocytes in the substantia nigra following LPS stimulation [50, 57]. We therefore sought to determine whether CD44 expression was altered in the substantia nigra of PD patients. By data mining publicly available human microarray data, we found that PD patients expressed significantly higher CD44 levels compared to age-matched controls (Fig. 1c). Similarly, utilizing the acute MPTP model, we found that CD44 levels are significantly higher at both 2 and 7 days following injection (Fig. 1d). Immunofluorescent microscopy confirmed the gene expression results, demonstrating increased CD44 protein levels in the SN with MPTP treatment, and GFAP-positive astrocytes contained twofold higher CD44 protein levels following MPTP compared to saline-treated animals (Fig. 1f). Similarly, when astrocytes were exposed to the CM treatment, we observed higher levels of CD44. These data, coupled with the previously published data with LPS, demonstrate that the increased CD44 levels likely play a role in neuroinflammation by regulating the response to GPNMB.

The hyaluranan receptor CD44 has been reported to play a role in peripheral inflammation [58]. There are conflicting reports, with some studies demonstrating that CD44 blocking antibodies can reduce inflammation, whereas others found that CD44 knockouts have increased inflammation [26, 59]. One study found that CD44 signaling could attenuate toll-like receptor (TLR) activation-mediated inflammation in bone marrow-derived macrophages [23]. To address the role of CD44 in mediating the effects of GPNMB in astrocytes, we utilized isolated primary mouse astrocytes from CD44 KO animals. These experiments determined that knockout of CD44 completely abolished the rGPNMB-mediated attenuation of the astrocyte inflammatory response. This included gene expression of the pro-inflammatory factors IL-6 and gp91phox and the anti-inflammatory factors arginase-1 and IGF-1. The CM-induced ROS and NO levels, along with iNOS expression, similarly showed no change when co-treated with rGPNMB in CD44 KO astrocytes. Taken together, these are the first set evidence directly demonstrating that CD44 mediates the GPNMB-induced attenuation of inflammation in cultured astrocytes. Further study is needed to define the mechanisms responsible for CD44 attenuation of inflammation in astrocytes, although the NFκB signaling pathway was suggested to play a role in CD44 regulation of the inflammatory response of bone marrow macrophages [23]. This is especially true since the CD44 KO mouse strain used in this study contains global knockout of CD44 and could potentially have some effects on development [60], although the astrocyte cultures in this study contained no overt abnormalities. It should also be noted that the recombinant GPNMB protein used in the study was a chimera Fc protein, and astrocytes express Fc receptors [61, 62]. However, since the CD44 KO astrocytes completely failed to respond to the recombinant GPNMB it is unlikely that the Fc portion of the GPNMB recombinant protein contributed to this effect.

## Conclusions

With the recent finding that inflammatory microglia can activate astrocytes to become neurotoxic [44], it is becoming clear that the astrocyte inflammatory status is important in neurodegenerative diseases, including PD. This study demonstrates a new pathway that can regulate the astrocyte inflammatory response, with GPNMB activating the CD44 receptor leading to an anti-inflammatory phenotype in cultured astrocytes. Although the role of this pathway in vivo remains to be established, our data provide additional mechanistic insight into the previously reported neuroprotective effects observed in animal models of ischemia and ALS. Additionally, the presence of CD44 on other cell types, including dopamine neurons [57] and microglia [52], suggests that GPNMB may represent a novel multi-target therapeutic approach to treat neurodegenerative diseases, including PD.

## Abbreviations

ALS: Amyotrophic lateral sclerosis
CM: Cytokine mix
FBS: Fetal bovine serum
GEO: Gene expression omnibus
GFAP: Glial firbrillary acidic protein
GPNMB: Glycoprotein non-metastatic melanoma protein B
IFNγ: Interferon gamma
IGF-1: Insulin growth factor 1
IL-1β: Interleukin 1 beta
IL-4: Interleukin-4
IL-6: Interleukin-6
iNOS: Inducible nitric oxide synthase
LPS: Lipopolysaccharide
MPTP: 1-Methyl-4-phenyl-1,2,3,6-tetrahydropyridine
NO: Nitric oxide
PD: Parkinson’s disease
PMA: Primary mouse astrocytes
qPCR: Quantitative polymerase gene expression
rGPNMB: Recombinant GPNMB
ROS: Reactive oxygen species
SN: Substantia nigra
SNP: Single nucleotide polymorphism
TNFα: Tumor necrosis factor alpha
